# Supplementary material for: The relationship between hyperglycaemia on admission and patient outcome is modified by hyperlactatemia and diabetic status: a retrospective analysis of the eICU collaborative research database
Source: Sci Rep. 2023 Sep 21;13:15692. doi: 10.1038/s41598-023-43044-7 (PMC10514185; doi:10.1038/s41598-023-43044-7)
Supplement: Supplementary file 1 — Supplementary Information. [file 41598_2023_43044_MOESM1_ESM.pdf]

The relationship between hyperglycaemia on admission and patient outcome is modified by hyperlactatemia and diabetic status: a retrospective analysis of the eICU collaborative research database

Oisin Fitzgerald, Oscar Perez-Concha, Blanca Gallego-Luxan, Lachlan Rudd, Louisa Jorm

Appendix A: Additional results

Descriptive statistics 2
Missingness and imputation models 4
Model selection 7
Model interpretation 12

Descriptive statistics

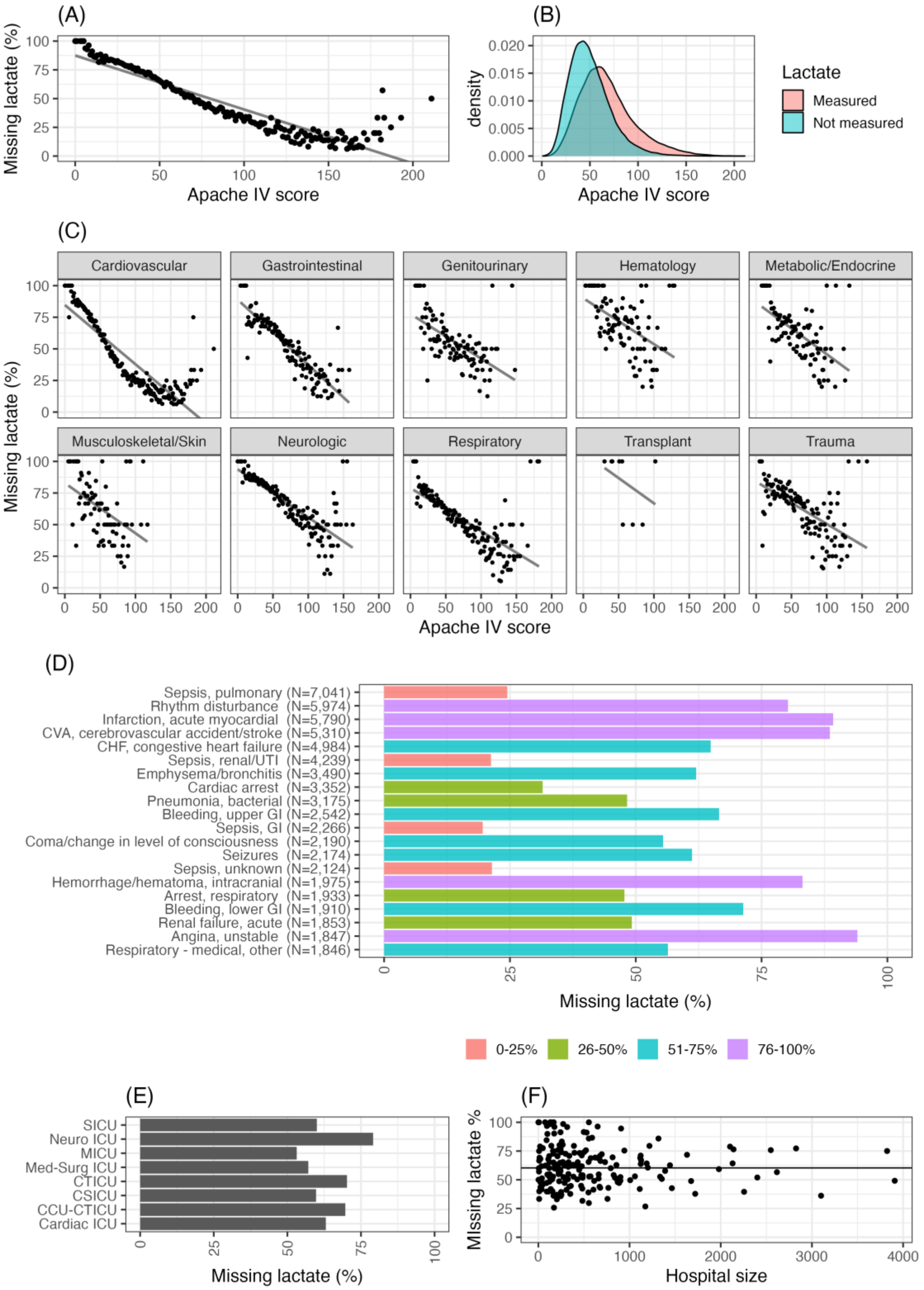

Figure A1. Characterisation of ICU stays with and without a blood lactate measurement. A) APACHE-IVa score and percentage of missing lactate. B) Distribution of APACHE-IVa score by indication of missing lactate. C) APACHE-IVa score and percentage of missing lactate by admission organ system. D) Top 20 admission diagnoses and percentage of missing lactate. E) ICU type and percentage of missing lactate. F) Hospital size and percentage of missing lactate.

Table A1. *Alternative version of Table 1 using the XGBoost weights.*

	**Overall**	**Weighted**	**Lactate measurement**	
			**Available**	**Not available**
Patients (N)	104,867	42,540	42,540	64,057
Hospitals	189	189	189	189
Patient characteristics at ICU admission				
ICU stays (N)	109,349	43,378	43,378	65,971
Age (years) (mean (SD))	64(17)	63 (17)	64 (17)	63 (17)
Gender: female (%)	54	53	53	54
BMI (kg/m^2) (mean (SD))	28.7 (7.7)	28.7 (7.8)	28.7 (8.0)	28.9 (7.6)
Diabetic (%)	21.9	22	22.9	21.3
Ethnicity (%)				
Caucasian	76.7	76.9	77.3	76.2
African American	11.8	12.3	12.1	11.6
Hispanic	4.0	3.2	3.3	4.4
Asian	1.4	1.4	1.4	1.4
Other/unknown	5.5	5.5	5	5.9
ICU type (%)				
Cardiac ICU	7.8	7.0	7.3	8.2
CCU-CTICU	8.3	8.4	6.4	9.6
CSICU	2.0	2.4	2.0	2.0
CTICU	1.9	2.3	1.4	2.2
Med-Surg ICU	56.7	56.1	61.3	53.7
MICU	10.3	10.7	12.2	9.1
Neuro ICU	7.5	7.7	3.9	9.8
SICU	5.4	5.5	5.5	5.4
ICU admission type				
Operative	2.8	2.8	2.8	2.9
Admission diagnosis of sepsis (%)				
Initial 24h of ICU stay				
Glasgow coma score (mean (SD))	12.8 (3.7)	12.7 (3.7)	11.7 (4.3)	13.4 (3.2)
APACHE-IVa score (mean (SD))	57 (26)	57 (26)	68 (28)	50 (21)
Glucose measures in first 24h				
Initial glucose (mg/dL) (mean (SD))	145 (74)	147 (75)	153 (84)	140 (65)
Matched glucose (mg/dL) (mean (SD))	143 (57)	146 (59)	151 (66)	137 (48)
Mean glucose (mg/dL) (mean (SD))	141 (51)	142 (50)	146 (54)	137 (48)
Maximum glucose (mg/dL) (mean (SD))	182 (98)	187 (100)	199 (111)	170 (87)
Glucose < 80 mg/dL (%)	15	16	19	12
Glucose < 50 mg/dL (%)	3	3	4	2
No glucose measurement in first 24 hours	2,099	128	128	1,971
Lactate measures in first 24h				
Initial lactate (mmol/L) (mean (SD))	n/a	2.27 (2.22)	2.55 (2.58)	n/a
Matched lactate (mmol/L) (mean (SD))	n/a	2.49 (2.41)	2.80 (2.73)	n/a
Mean lactate (mmol/L) (mean (SD))	n/a	2.21 (1.96)	2.49 (2.32)	n/a
Maximum lactate (mmol/L) (mean (SD))	n/a	2.68 (2.70)	3.08 (3.15)	n/a
Other lab measures in first 24h				
Mean total bilirubin (mg/dL) (mean (SD))	1.06 (2.21)	1.03 (2.06)	1.22 (2.49)	0.92 (1.91)
Mean creatinine (mg/dL) (mean (SD))	1.62 (1.78)	1.63 (1.82)	1.85 (1.89)	1.46 (1.67)
Interventions in first 24h				
Treated with insulin (%)	30.3	34.5	37.7	25.4
Ventilated (%)	32	33	45	24
Intubated (%)	24	24	36	16
Patient outcomes				
Hospital mortality (%)	10.3	11.0	16.2	6.5
ICU mortality (%)	6.3	6.8	10.7	3.4
Length of ICU stay (hours) (mean (SD))	79 (104)	82 (102)	95 (118)	68 (92)

Missingness and imputation models

Missingness models

Table A2. Missing blood lactate logistic regression model (exp. = exponentiated).

Variable	Coefficient	Standard error	Lower 95%	Upper 95%
Intercept	1.9285	0.0412	1.8479	2.0092
APACHE diagnosis: sepsis	-3.0040	0.0580	-3.1177	-2.8903
APACHE IVa score	-0.0255	0.0004	-0.0263	-0.0247
APACHE IVa score : Sepsis	0.0145	0.0008	0.0129	0.0162
Age	0.0114	0.0005	0.0105	0.0124
Operative admission	0.1711	0.0428	0.0872	0.2550
Ventilated	-0.2916	0.0255	-0.3415	-0.2417
Intubated	-0.3252	0.0286	-0.3813	-0.2692
APACHE diagnosis organ system				
Gastrointestinal	-0.6809	0.0263	-0.7325	-0.6294
Genitourinary	-0.9256	0.0444	-1.0127	-0.8385
Hematology	-0.4828	0.0723	-0.6245	-0.3411
Metabolic/Endocrine	-0.6891	0.0509	-0.7888	-0.5894
Musculoskeletal/Skin	-0.8738	0.0872	-1.0447	-0.7030
Neurologic	-0.0371	0.0240	-0.0842	0.0100
Respiratory	-0.5382	0.0224	-0.5821	-0.4943
Transplant	-1.2388	0.4179	-2.0578	-0.4198
Trauma	-0.3687	0.0380	-0.4433	-0.2942
ICU type				
CCU-CTICU	0.1979	0.0364	0.1266	0.2692
CSICU	-0.1922	0.0562	-0.3024	-0.0820
CTICU	0.1269	0.0596	0.0100	0.2438
Med-Surg ICU	-0.0522	0.0274	-0.1059	0.0014
MICU	-0.0203	0.0336	-0.0861	0.0455
Neuro ICU	0.5352	0.0411	0.4547	0.6157
SICU	-0.0546	0.0398	-0.1326	0.0234

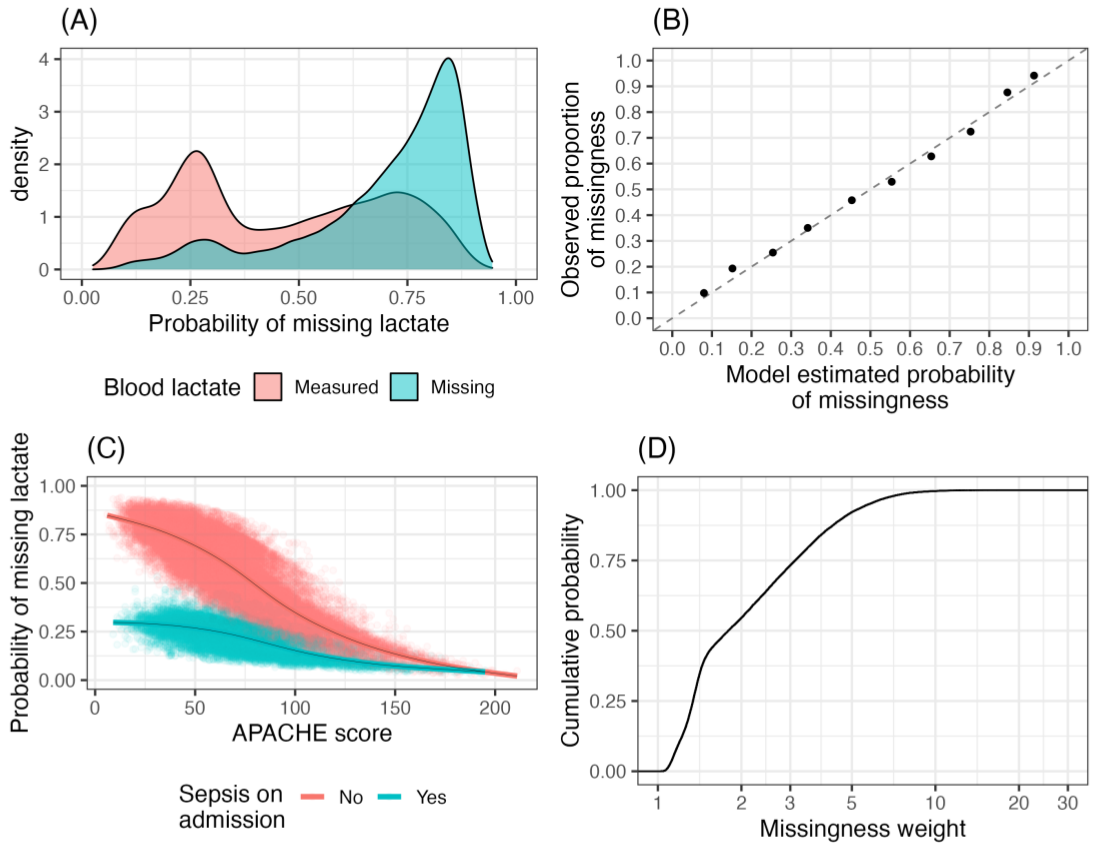

Figure A2. Lactate missingness model results. A) Distribution of model probability of missingness by actual missingness category, an indication of model discriminatory ability. B) Model calibration curve. Each point includes 95% CI (largely not visible due to large sample size). C) Estimated probability of missingness by APACE-IVa score and sepsis diagnosis. C) Distribution of missingness weights

The XGBoost missingness model (Table A2) has an AUC-ROC of 0.823.

Table A3. Variable importance (top 20) for the XGBoost missingness model

Feature	Gain	Gain SD
Sepsis	0.2308	0.0018
Apache IVa score	0.1279	0.0113
Bicarbonate	0.1062	0.0021
Intubated	0.0570	0.0120
Calcium	0.0537	0.0015
Chloride	0.0452	0.0024
Potassium	0.0381	0.0013
BMI	0.0386	0.0009
Sodium	0.0388	0.0013
Creatinine	0.0331	0.0016
BUN	0.0305	0.0017
Age	0.0252	0.0006
Ventilated	0.0242	0.0034
CVA	0.0150	0.0005
MI	0.0145	0.0006
GCS	0.0122	0.0022
Pneumonia	0.0076	0.0004
Neuro ICU	0.0079	0.0007
Unstable angina	0.0062	0.0004

Imputation models

The linear regression (GAM) blood lactate imputation model (Table A3) had a RMSE of 2.513, while the XGBoost model (Table A4) had a RMSE of 2.412 mmol/L.

Table A4. *Blood lactate linear GAM model*

Variable	Coefficient	Standard error	Lower 95%	Upper 95%
Intercept	3.7993	0.0766	3.6490	3.9495
APACHE diagnosis: sepsis	-0.0836	0.0359	-0.1541	-0.0132
Age	-0.0113	0.0008	-0.0129	-0.0097
Operative admission	-0.1067	0.0780	-0.2595	0.0461
Ventilated	-0.0847	0.0436	-0.1703	0.0008
Intubated	0.2956	0.0477	0.2021	0.3890
APACHE diagnosis organ system				
Gastrointestinal	-0.0456	0.0497	-0.1431	0.0519
Genitourinary	-0.7250	0.0780	-0.8779	-0.5722
Hematology	-0.4560	0.1401	-0.7305	-0.1815
Metabolic/Endocrine	-0.0769	0.0963	-0.2657	0.1120
Musculoskeletal/Skin	-1.0303	0.1577	-1.3394	-0.7212
Neurologic	-0.4820	0.0474	-0.5749	-0.3890
Respiratory	-0.8597	0.0412	-0.9405	-0.7789
Transplant	-2.0875	0.5509	-3.1673	-1.0077
Trauma	-0.1058	0.0744	-0.2516	0.0400
ICU type				
CCU-CTICU	-0.0951	0.0656	-0.2236	0.0334
CSICU	-0.2392	0.0960	-0.4274	-0.0511
CTICU	-0.0791	0.1114	-0.2975	0.1394
Med-Surg ICU	-0.0383	0.0475	-0.1313	0.0548
MICU	-0.1227	0.0567	-0.2338	-0.0117
Neuro ICU	-0.2274	0.0778	-0.3798	-0.0750
SICU	-0.1486	0.0697	-0.2852	-0.0119
Spline terms				
APACHE IVa score	n/a	$p < .001$	n/a	n/a

Table A5. Variable importance (top 20) for the XGBoost imputation model

Feature	Gain	Gain SD
Bicarbonate	0.2451	0.0067
Apache IVa score	0.2352	0.0050
Chloride	0.1220	0.0066
Sodium	0.1018	0.0068
BUN	0.0567	0.0029
APACHE diagnosis: Cardiac Arrest	0.0519	0.0033
Creatinine	0.0458	0.0039
Potassium	0.0216	0.0014
Calcium	0.0214	0.0033
BMI	0.0178	0.0011
Age	0.0146	0.0018
APACHE diagnosis: Seizure	0.0111	0.0018
Intubated	0.0074	0.0015
APACHE diagnosis: Sepsis	0.0068	0.0006
APACHE diagnosis: Upper GI bleed	0.0048	0.0012
GCS (verbal)	0.0038	0.0005
GCS	0.0037	0.0009
oobventday1	0.0018	0.0013
APACHE diagnosis: GI bleed	0.0012	0.0004

Model selection

The following tables (A2-6) graph the cross-validation results across various sensitivity analysis except Table A3 which repeats the results in Table 3.

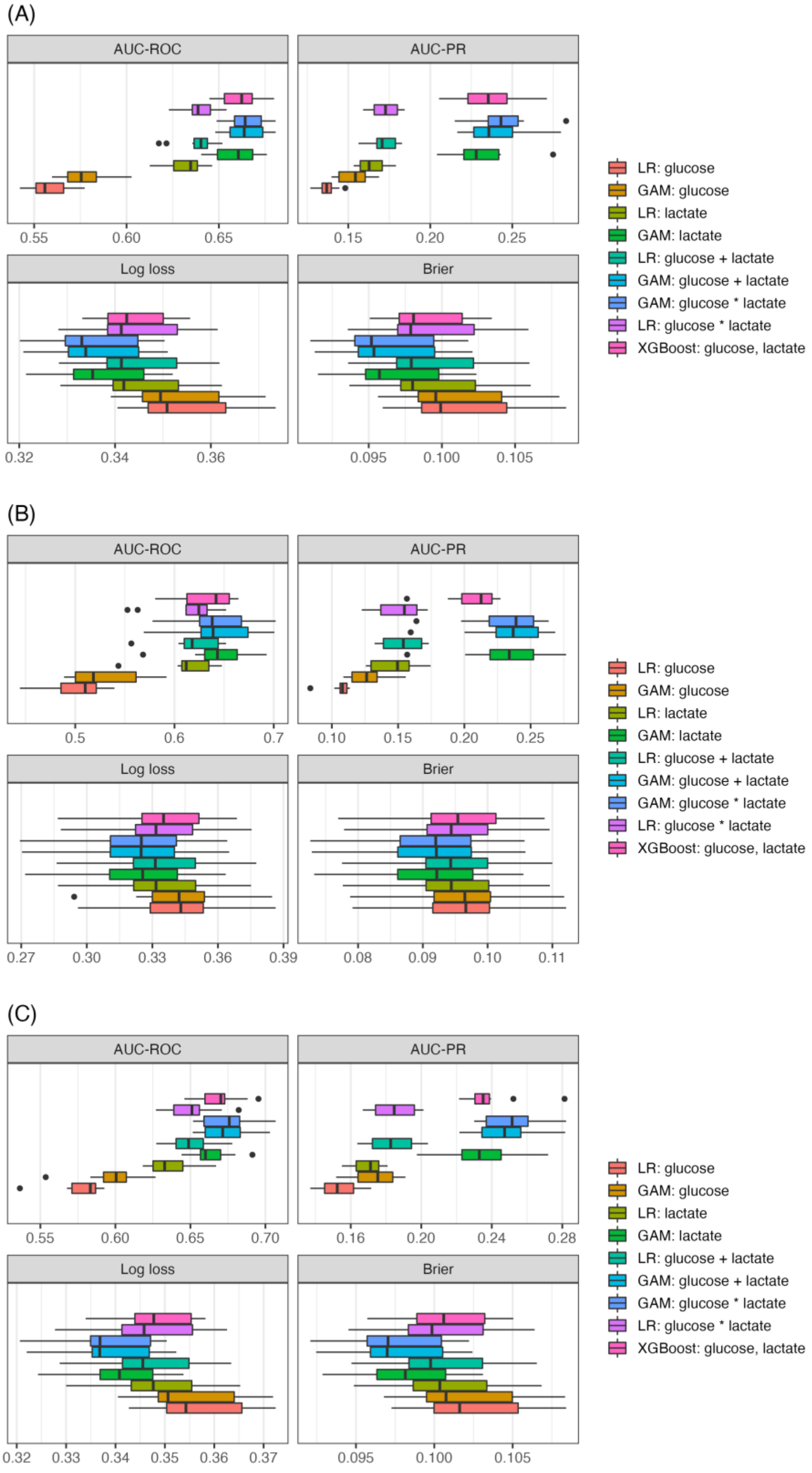

Figure A3. Weighted cross-validation results using logistic regression generated weights (A) All patients (B) Diabetics. (C) Non-diabetics.

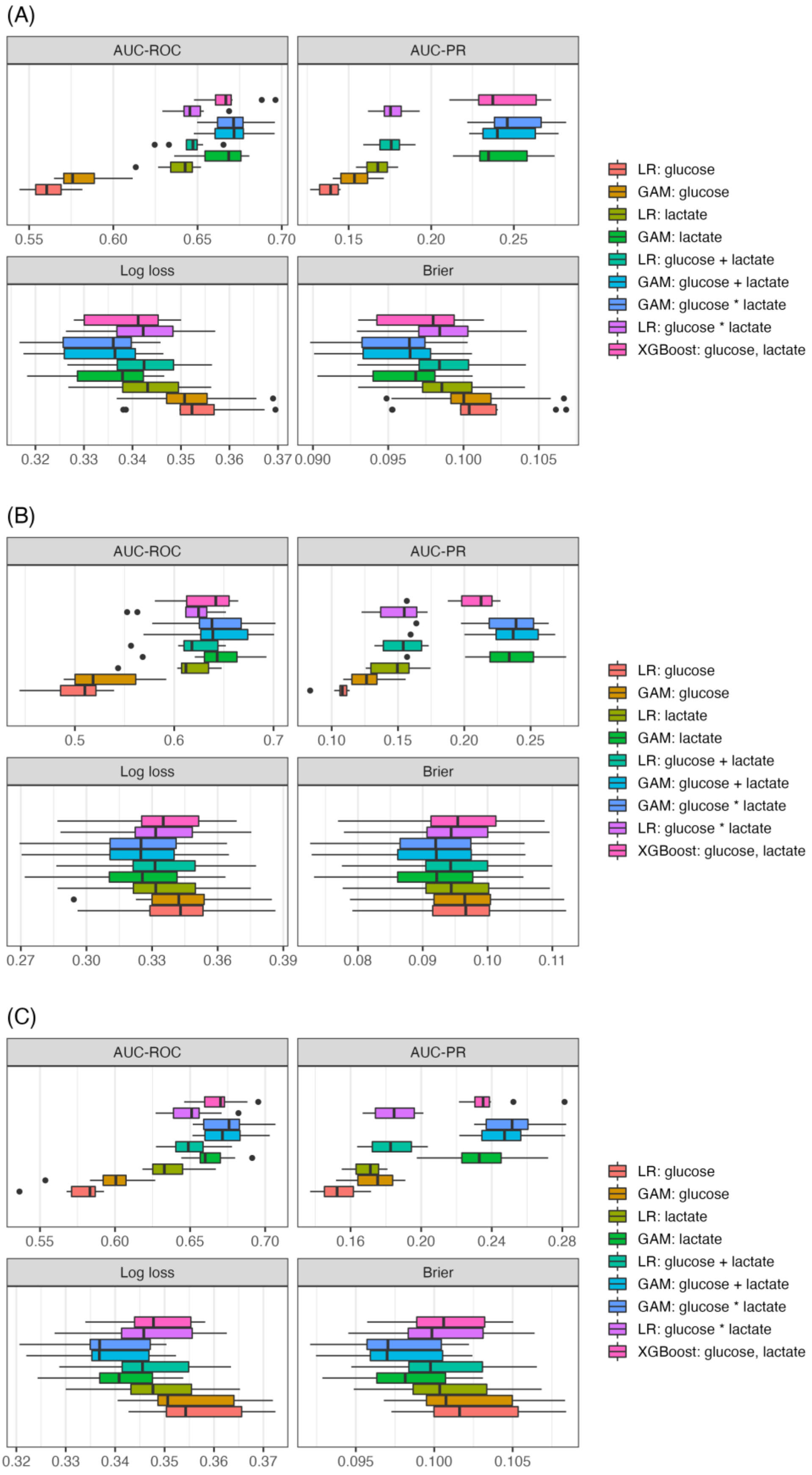

Figure A4. Weighted cross-validation results using XGBoost generated weights (A) All patients (B) Diabetics. (C) Non-diabetics.

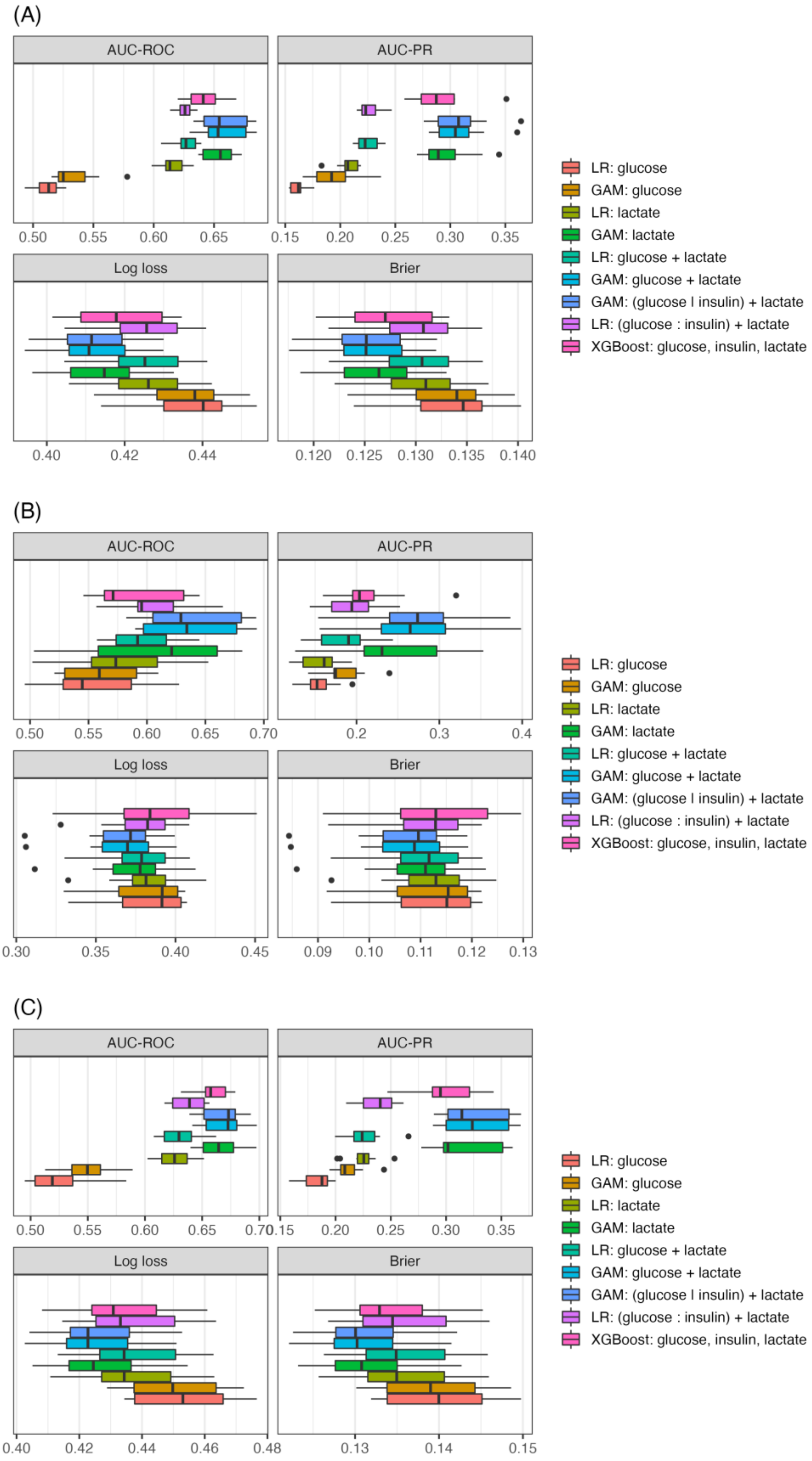

Figure A5. Weighted cross-validation results for patients with an admission diagnosis of Sepsis using averaged weights (A) All patients (B) Diabetics. (C) Non-diabetics.

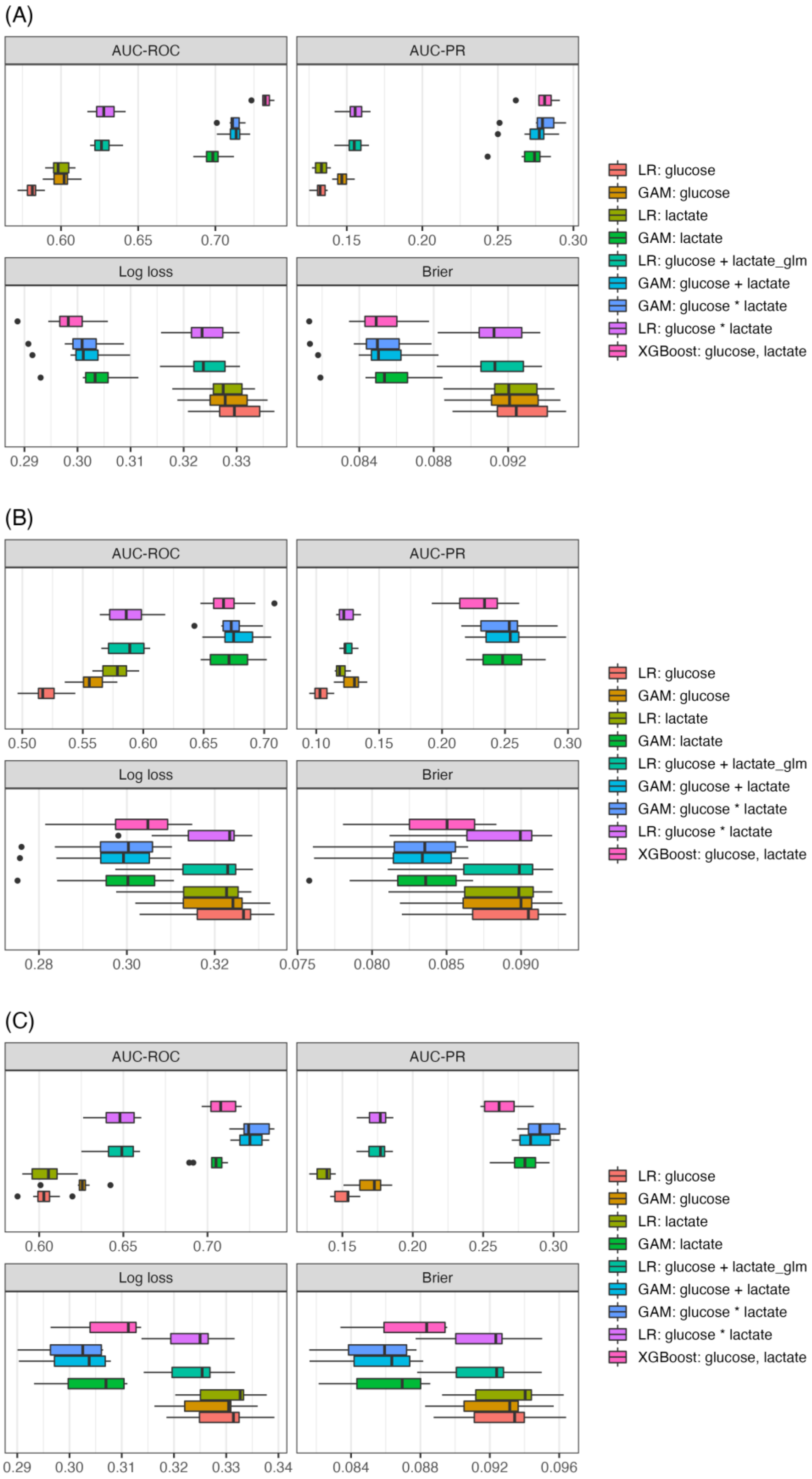

Figure A6. Cross-validation results with missing lactate values imputed using the linear GAM described in Table A3 (A) All patients (B) Diabetics. (C) Non-diabetics.

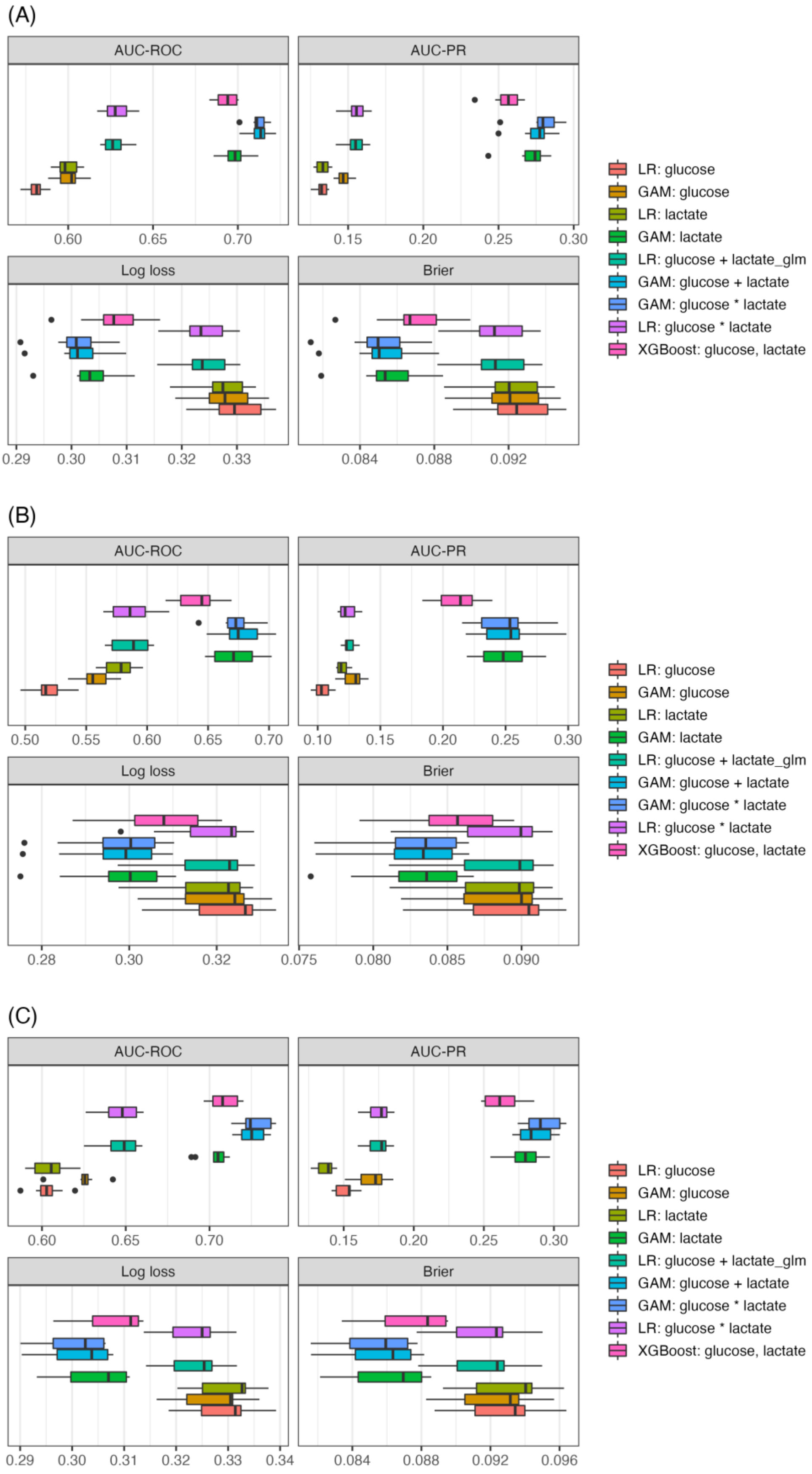

Figure A7. Cross-validation results with missing lactate values imputed using the XGBoost model described in Table A4 (A) All patients (B) Diabetics. (C) Non-diabetics.

Model interpretation

The following tables (A7-8) graph the impact of blood glucose on hospital mortality.

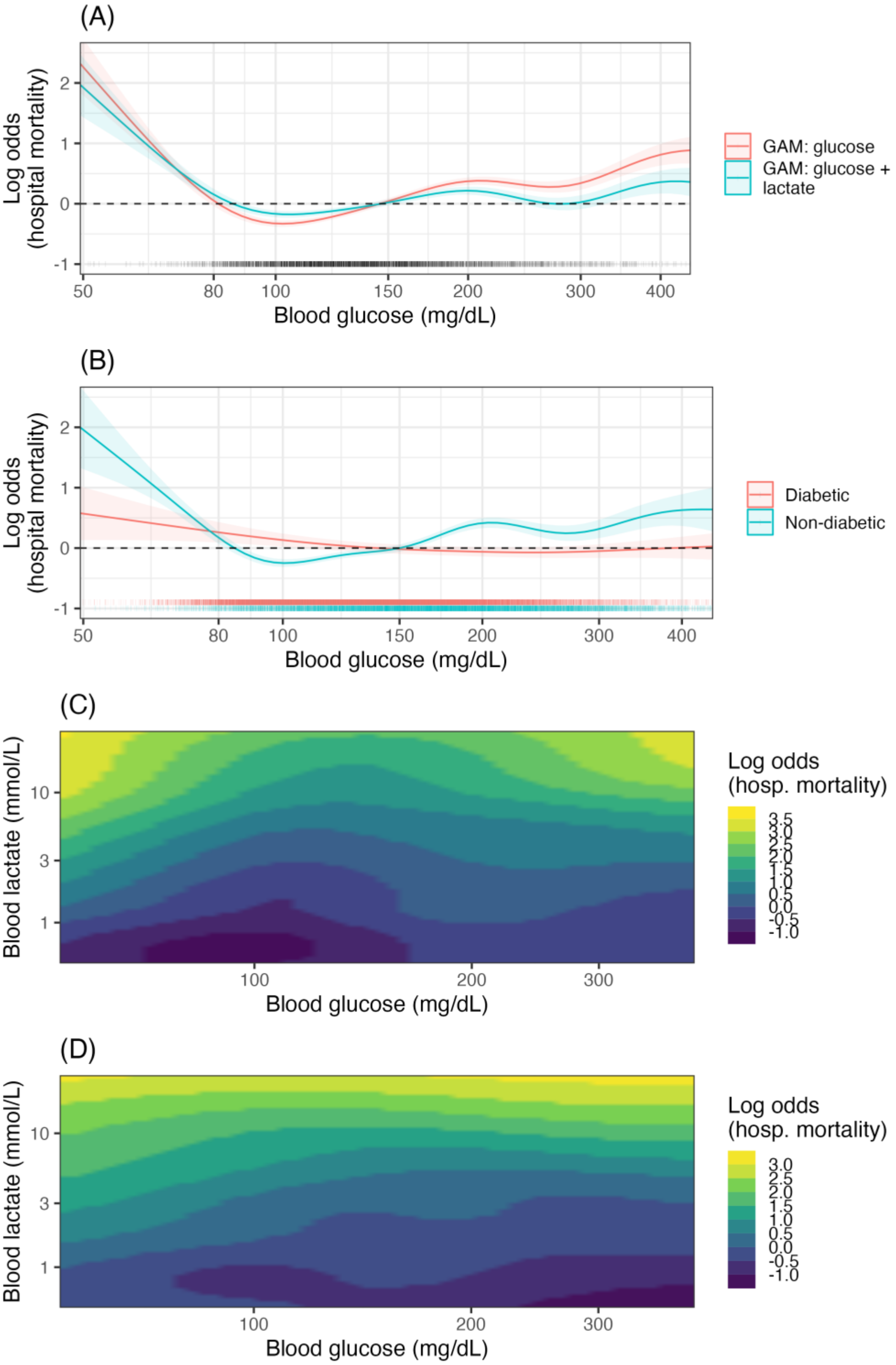

Figure A8. GAM model partial effects (log odds scale) for several GAM models using the XGBoost generated weights. A) The impact of adjustment for lactate on the partial effect of glucose. B) The partial effects of glucose for diabetics and non-diabetics *GAM: glucose + lactate.* C) The 2D spline interaction effect between blood glucose and lactate levels for non-diabetics *GAM: (glucose : lactate | DM = 0).* D) The 2D spline interaction effect between blood glucose and lactate levels for diabetics *GAM: (glucose : lactate | DM = 1).*

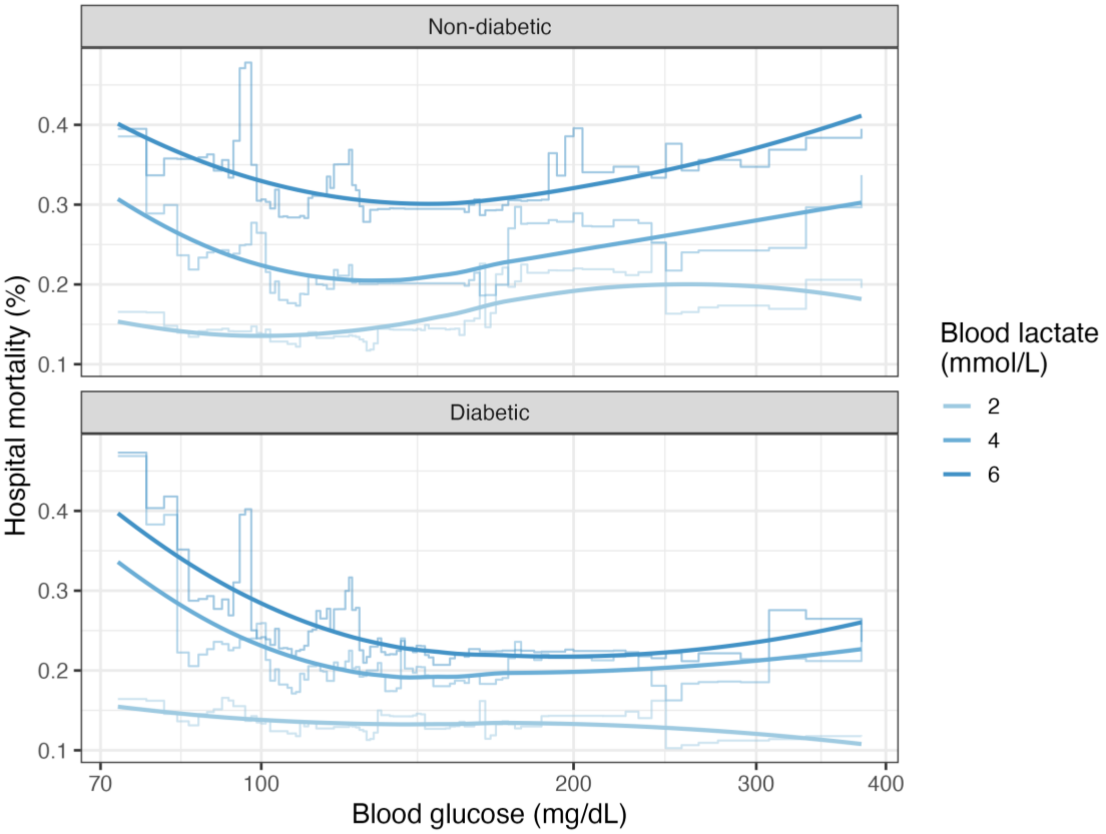

Figure A9. Nonparametric conditional estimates of mortality risk by blood glucose and blood lactate levels stratified by diabetic status using XGBoost

Table A6. Logistic regression model with hospital mortality as outcome and blood glucose and blood lactate interaction terms

Variable	Coefficient	Standard error	Statistic	p value
(Intercept)	-3.081	0.064	-48.026	0.000
Blood glucose (126-137]	0.307	0.139	2.217	0.027
Blood glucose (137-148]	0.359	0.147	2.446	0.014
Blood glucose (148-162]	0.430	0.165	2.602	0.009
Blood glucose (162+)	0.727	0.125	5.792	0.000
Blood lactate (1-1.3]	0.438	0.082	5.312	0.000
Blood lactate (1.3-1.7]	0.543	0.085	6.351	0.000
Blood lactate (1.7-2.3]	0.656	0.079	8.330	0.000
Blood lactate (2.3+)	1.442	0.064	22.635	0.000
Blood glucose (126-137]:Blood lactate (1-1.3]	-0.374	0.205	-1.824	0.068
Blood glucose (137-148]:Blood lactate (1-1.3]	0.078	0.201	0.385	0.700
Blood glucose (148-162]:Blood lactate (1-1.3]	0.058	0.222	0.264	0.792
Blood glucose (162+):Blood lactate (1-1.3]	-0.141	0.174	-0.811	0.418
Blood glucose (126-137]:Blood lactate (1.3-1.7]	-0.108	0.203	-0.532	0.595
Blood glucose (137-148]:Blood lactate (1.3-1.7]	-0.126	0.216	-0.583	0.560
Blood glucose (148-162]:Blood lactate (1.3-1.7]	-0.266	0.232	-1.143	0.253
Blood glucose (162+):Blood lactate (1.3-1.7]	-0.194	0.170	-1.140	0.254
Blood glucose (126-137]:Blood lactate (1.7-2.3]	-0.257	0.186	-1.386	0.166
Blood glucose (137-148]:Blood lactate (1.7-2.3]	-0.184	0.197	-0.934	0.350
Blood glucose (148-162]:Blood lactate (1.7-2.3]	-0.441	0.214	-2.066	0.039
Blood glucose (162+):Blood lactate (1.7-2.3]	-0.065	0.152	-0.430	0.667
Blood glucose (126-137]:Blood lactate (2.3+)	-0.395	0.156	-2.531	0.011
Blood glucose (137-148]:Blood lactate (2.3+)	-0.493	0.165	-2.986	0.003
Blood glucose (148-162]:Blood lactate (2.3+)	-0.505	0.179	-2.818	0.005
Blood glucose (162+):Blood lactate (2.3+)	-0.391	0.133	-2.926	0.003
